# 

**Published:** 1881-07

**Authors:** 


### CONTENTS FOR JULY, 1881.

ORIGINAL COMMUNICATIONS.

ART. I.—Observations on Yellow Fever and its Treatment. By HARVEY L. BYRD, A. M., M. D. .......... 271
ART. II.—Nitrous Oxide Gas. By LAURENCE TURNBULL, M. D. .......... 387

EDITORIAL DEPARTMENT.

Free Baths for Baltimore .......... 391
New Medical Building for Baltimore .......... 392
Quinine in Tetanus .......... 393
Mistletoe as an Anti-Hemorrhagic .......... 394
Malpractice Suit .......... 395
John H. Griscom .......... 395
The International Medical Congress .......... 396
Cremation of Dr. Colletti .......... 396
The New York Street Cleaning Bill .......... 396
Sunnyside .......... 397
Acne Punctata .......... 397
Meetings of Dental Societies in July .......... 397

BIBLIOGRAPHICAL DEPARTMENT.

The Quarterly Epitome of Practical Medicine and Surgery .......... 398
The Indiana Medical Reporter .......... 398
The Pathologist .......... 399
Chest Drainage in Empyema .......... 399
Shortening of Limbs after Fracture .......... 399

THERAPEUTIC PEARLS AT RANDOM STRUNG .......... 400
SELECTIONS .......... 402-420

POPULAR SCIENCE DEPARTMENT.

The Smoke and Fog of London .......... 421
The Filth of the Middle Ages .......... 422
The Anæsthetic Action of Chlorine .......... 424
Ovarian Tumor Treated by Incision and Drainage .......... 424
Pains and Anodynes .......... 424
Insanity and Uterine Diseases .......... 426
The Hygiene of Food .......... 426
Macadamized Streets .......... 429
Photographs in Natural Colors .......... 430
The Quality of Infection .......... 431
Palæolithic Osteology .......... 431
To Avoid Infection .......... 433
Consumption in Cows .......... 433
That Bedroom .......... 434
Electric Light Notes .......... 435
Parasite in Lepra .......... 437
Fireless Locomotives .......... 437
Resorcine .......... 439
Concentrating or Storing up Electricity .......... 439
Telegraph Cable in Sewers .......... 441
New Remedy for Baldness .......... 442
HYMENEAL. NECROLOGICAL .......... 442

